# Mechanisms of Aluminum Toxicity Impacting Root Growth in Shatian Pomelo

**DOI:** 10.3390/ijms252413454

**Published:** 2024-12-15

**Authors:** Jingfu Yan, Wenbo Zhu, Dongshen Wu, Xinya Chen, Shaoxia Yang, Yingbin Xue, Ying Liu, Ying Liu

**Affiliations:** 1 Department of Agronomy, College of Coastal Agricultural Sciences, Guangdong Ocean University, Zhanjiang 524088, China; y1229082423@163.com (J.Y.); puting2833@126.com (D.W.); 13760608838@163.com (X.C.);; 2 Qinghai Provincial Key Laboratory of Adaptive Management on Alpine Grassland, Qinghai Academy of Animal and Veterinary Sciences, Qinghai University, Xining 810010, China; 3 Key Laboratory of Superior Forage Germplasm in the Qinghai-Tibetan Plateau, Qinghai Academy of Animal and Veterinary Sciences, Qinghai University, Xining 810010, China

**Keywords:** *Citrus maxima* ‘Shatian Yu’, Al toxicity stress, roots, physiological mechanism

## Abstract

Aluminum (Al) toxicity in acidic soils poses significant challenges to crop growth and development. However, the response mechanism of Shatian pomelo (*Citrus maxima* ‘Shatian Yu’) roots to Al toxicity remains poorly understood. This study employed root phenotype analysis, physiological response index measurement, root transcriptome analysis, and quantitative PCR (qPCR) validation to investigate the effects of Al toxicity on Shatian pomelo roots. The findings revealed that Al toxicity inhibited root growth and development, resulting in reduced root biomass, total root length, total root surface area, root volume, average root diameter, and root tip count. Antioxidant enzyme activities (peroxidase, superoxide dismutase, ascorbate peroxidase, and catalase activity) and soluble protein content increased with rising Al toxicity, whereas malondialdehyde content initially increased and then declined. Additionally, Al toxicity stress increased Al (1439.25%) content and decreased boron (B, 50.64%), magnesium (Mg, 42.04%), calcium (Ca, 46.02%), manganese (Mn, 86.75%), and iron (Fe, 69.92%) levels in the roots. RNA sequencing (RNA-seq) analysis identified 3855 differentially expressed genes (DEGs) between 0 mmol/L Al (control) and 4 mmol/L Al (Al toxicity) concentrations, with 1457 genes up-regulated and 2398 down-regulated, indicating a complex molecular regulatory response. The qPCR results further validated these findings. This study elucidates the response mechanisms of Shatian pomelo roots to Al toxicity stress, providing insights into the regulatory pathways involved. The findings offer valuable reference points for breeding Al-resistant Shatian pomelo varieties. The results of this study provide important genetic tools and technical support for the screening and breeding of highly resistant varieties of Shatian pomelo. On the one hand, by detecting the key indexes (such as antioxidant enzyme activity and nutrient absorption capacity) of Shatian pomelo, varieties with excellent anti-Al toxicity characteristics can be selected. On the other hand, the Al-resistant genes identified in this study, such as *TFM1* and *ALERTFA0*, can be used to develop molecular markers, assisted marker breeding, or transgenic breeding to accelerate the breeding process of Al-resistant strains.

## 1. Introduction

With the intensification of industrialization, there has been a continuous and unreasonable discharge of acid gases into the atmosphere, resulting in severe acid deposition [1] and accelerating soil acidification [2]. The main planting areas of Shatian pomelo are concentrated in southern China [3]. The total area of acidic red soil in 15 southern provinces of China is 2.18 million km^2^, accounting for 22.7% of the total land area of the country [3]. Research indicates that the solubility of toxic Al^3+^ significantly increases when soil pH falls below 5.0, leading to a substantial rise in aluminum (Al) content in soil solutions [4]. Consequently, as soil acidification continues, Al increasingly emerges as the primary metal element that limits plant growth [5]. Al is a prevalent metallic element in the Earth’s crust, constituting approximately 8.7% of its total mass [6]. In acidic soils, Al is present in various forms, including trivalent Al ions, Al hydroxide ions, and dihydroxy-Al ions [7]. Al ions can influence plant growth and development in multiple ways [8]. When subjected to high concentrations of Al toxicity, crops initially exhibit growth retardation, significantly diminishing crop quality [9]. Research indicates that the dry mass of leaves (DML) and roots (DMR) in maize (*Zea mays*) under Al toxicity stress decreased by 2.35% and 11.63%, respectively, compared to the control group, reflecting inhibited biomass accumulation [10]. Prolonged exposure to Al toxicity results in the continuous accumulation of Al ions in plant root cell walls, which impedes cell division and elongation, thus inhibiting overall root development [11]. Furthermore, studies revealed that root tip cells of *Eucalyptus* experience significant damage under Al toxicity, with root crown cells cracking and becoming soft [12]. The excessive accumulation of Al can also induce the production of reactive oxygen species (ROS) in plants, resulting in oxidative stress. This condition leads to membrane lipid peroxidation, compromising the functionality of organelles associated with the plasma membrane and disrupting normal physiological metabolism [13]. In response, the plant root system activates its antioxidant defense mechanism, primarily by enhancing the activity of key antioxidant enzymes such as catalase (CAT), superoxide dismutase (SOD), and peroxidase (POD) to mitigate the effects of accumulated ROS and free radicals under Al toxicity stress. This process helps reduce oxidative damage and maintain intracellular REDOX balance [14]. For instance, CAT and SOD activities in *Trifolium repens* increased by 172.97% and 84.48%, respectively, under Al stress compared to the normal growing conditions [15]. Additionally, Al ions can adhere to the phospholipid bilayer of the plasma membrane, destabilizing the membrane potential, inhibiting the proton removal activity of H^+^-adenosine triphosphatase (H^+^-ATPase), and affecting the transport of key ions, including K^+^, NH^4+^, Mg^2+^, and Ca^2+^ [16]. Research indicates that, compared to the control group, the Al concentration in the roots of *Neolamarckia cadamba* exposed to 400 μM AlCl_3_ sharply increased from 0 to 2140 μg/g on the first day. Additionally, calcium (Ca) concentrations decreased to 137 mg/g and 85 mg/g on the third and seventh days, respectively [8]. The levels of magnesium (Mg) and manganese (Mn) in root tips also significantly decreased on the first and third days, whereas iron ion concentrations underwent a marked decline by the third day [8].

RNA sequencing (RNA-seq) is a high-throughput sequencing technology that enables the detection of plant transcriptome information, thereby opening new avenues for plant research [17]. Recently, this technology has been successfully employed to analyze molecular regulatory mechanisms in various plant species, including rice (*Oryza sativa*), tobacco (*Nicotiana tabacum*), lingzhi (*Ganoderma lucidum*), poria cocos (*Wolfiporia cocos*), danshen (*Salvia miltiorrhiza*), and cape jasmine (*Catharanthus roseus*) [18].

Shatian pomelo (*Citrus maxima* ‘Shatian Yu’) is a significant fruit crop known for its high economic value [19]. For example, in 2023, the planting area of Shatian pomelo in Rong County, Guangxi Province, China, was about 37,889 acres, with an output of up to 360,000 tons and an output value of more than RMB 4 billion, and has become an important pillar of the local economy [19]. The fruit’s peel possesses various health benefits, strengthening the stomach, moistening the lungs, nourishing the blood, promoting wound healing, and serving as an excellent adjunct for managing sepsis and other diseases, etc. [20]. Recent studies have demonstrated that essential oil extracted from Shatian pomelo peel has been utilized across multiple important sectors, including in agricultural bacteriostasis, aromatherapy, and medical treatment and healthcare [21]. Research indicates that Al toxicity significantly inhibits root development in citrus crops, including grapefruit. For example, the taproot length and dry root weight of lemon (*Citrus* × *limon* Osbeck) plants under Al toxicity can be reduced by 2.3 times compared to normal plants, particularly in acidic soils, adversely affecting growth and yield [22]. Despite these findings, the molecular regulatory mechanisms governing Shatian pomelo root systems under Al toxicity stress remain unclear. This study employs high-throughput deep sequencing technology to analyze the transcriptome of Shatian pomelo roots subjected to Al toxicity stress.

Roots play a critical role in nutrient uptake for plants [23]. However, the response mechanisms of the Shatian pomelo root system to Al toxicity stress are still largely unknown, and further exploration of the physiological regulatory pathways is warranted. The primary aim of this study is to elucidate the response mechanisms of the Shatian pomelo root system to Al toxicity stress, including the changes in antioxidant enzyme activity, nutrient content, and gene expression in vivo under stress, etc., thereby providing a scientific basis for the development of new Shatian pomelo varieties that exhibit resistance to Al.

## 2. Results

### 2.1. Effects of Al Toxicity on Root Development of Shatian Pomelo Plants

**Figure 1:**
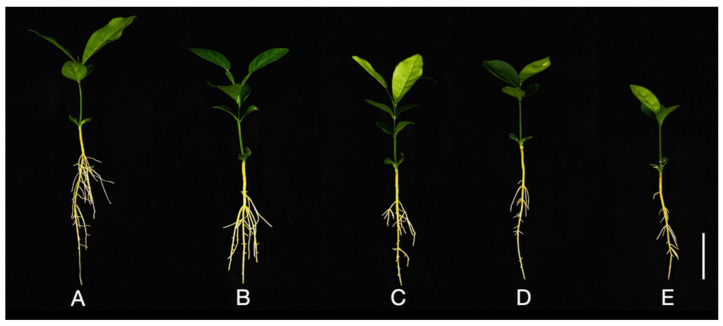
Phenotype of Shatian pomelo treated with different Al concentrations. Shatian pomelo was treated with different concentrations of Al for 20 days: (**A**) 0; (**B**) 1.0; (**C**) 2.0; (**D**) 4.0; (**E**) 8.0 mmol/L (bar = 5 cm).

**Figure 2:**
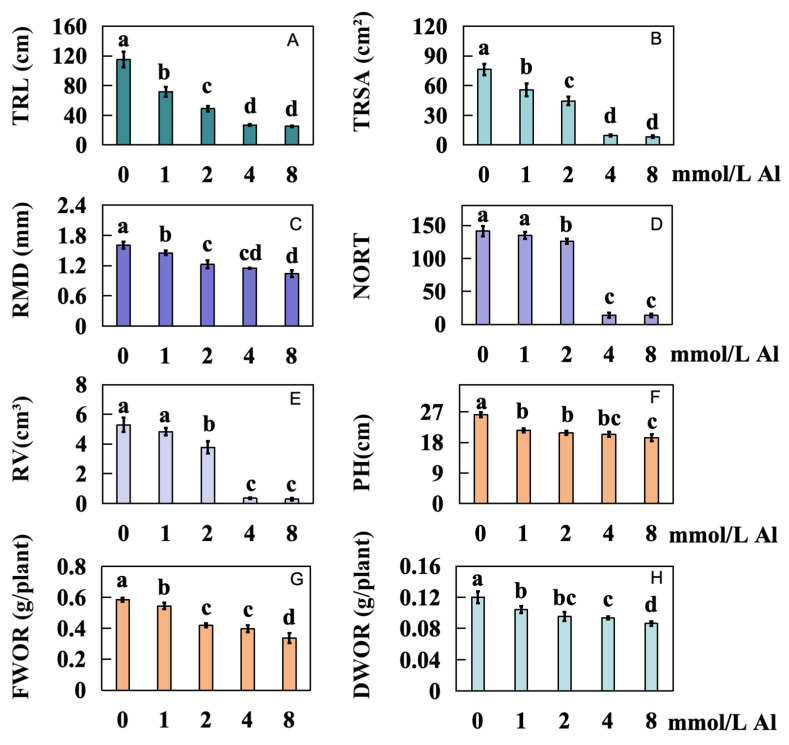
Effects of different concentrations of Al on root development of Shatian pomelo. The control group was treated with 0 mmol/L Al, while the experimental group was treated with 1.0, 2.0, 4.0, and 8.0 mmol/L Al. The Shatian pomelo plants were collected and measured after 20 days. (**A**) Total root length (TRL), (**B**) total root surface area (TRSA), (**C**) root mean diameter (RMD), (**D**) number of root tips (RORT), (**E**) root volume (RV), (**F**) plant height (PH), (**G**) fresh weight of root (FWOR); (**H**) dry weight of root (DWOR). The results are expressed as the mean and standard deviation of three repeated biological experiments. Waller–Duncan multiple comparison tests and a one-way analysis of variance were used to compare the significant differences between the control group and the treatment group. Different letters on the bar chart indicate significant differences between data (*p* < 0.05).

The root growth of Shatian pomelo was significantly inhibited under Al toxicity stress (Figure 1A–E). Compared to the control group with 0 mmol/L Al, increasing concentrations of exogenous Al (1.0, 2.0, 4.0, and 8.0 mmol/L) markedly decreased the total root length of Shatian pomelo by 37.78%, 57.56%, 76.62%, and 78.14%, respectively (Figure 2A). The total root surface area also decreased significantly by 27.19%, 41.79%, 87.54%, and 89.08%, respectively (Figure 2B). Additionally, the average diameter of the Shatian pomelo roots declined by 9.38%, 23.75%, 28.12%, and 35%, respectively (Figure 2C). The number of root tips diminished by 4.48%, 10.85%, 90.09%, and 90.33%, respectively (Figure 2D). Correspondingly, the total root volume experienced reductions of 8.70%, 28.73%, 93.38%, and 94.52%, respectively (Figure 2E). Plant height significantly decreased by 17.49%, 20.55%, 21.96%, and 26.01%, respectively (Figure 2F). The fresh root weight also decreased significantly by 6.74%, 28.38%, 32.10%, and 42.37%, respectively (Figure 2G). Finally, the dry root weight saw significant reductions of 13.40%, 20.65%, 22.15%, and 28.14%, respectively (Figure 2H).

### 2.2. Effects of Al Toxicity on Physiological Response Indexes of Shatian Pomelo Root System

**Figure 3:**
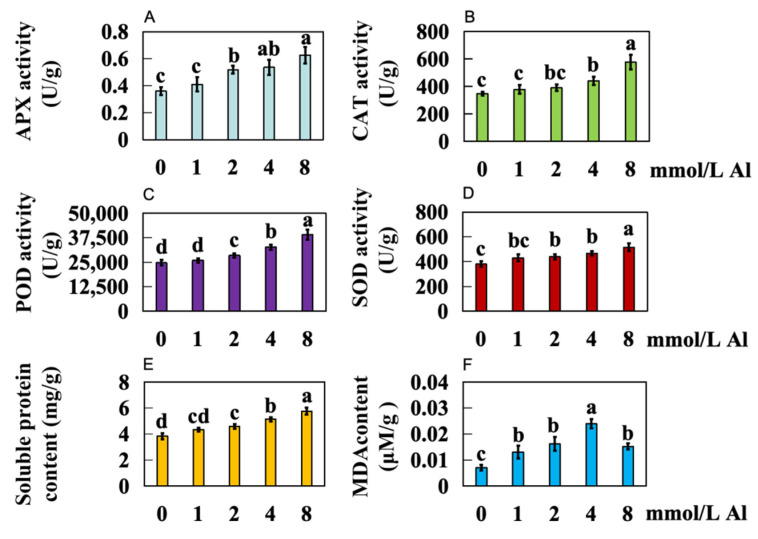
Results of physiological response indexes of Shatian pomelo root under Al toxicity stress. The Shatian pomelo plants were treated with Al concentrations of 1.0, 2.0, 4.0, and 8.0 mmol/L for 20 days, and the control group was treated with 0 mmol/L Al. Eight physiological indexes were determined, namely (**A**) APX (Ascorbate peroxidase) activity, (**B**) CAT (Catalase) activity, (**C**) POD (Peroxidase) activity, (**D**) SOD (Superoxide dismutase) activity, (**E**) soluble protein content, (**F**) Malondialdehyde (MDA) content. The results are expressed as the mean and standard deviation of three repeated biological experiments. Waller–Duncan multiple comparison tests and a one-way analysis of variance were used to compare the significant differences between the control group and the treatment group. Different letters on the bar chart indicate significant differences between the data (*p* < 0.05).

In comparison to the control group with 0 mmol/L Al, higher concentrations of exogenous Al led to increased activity of ascorbate peroxidase (APX) in the roots of Shatian pomelo in the experimental group (1.0, 2.0, 4.0, and 8.0 mmol/L Al), with activity increases of 13.89%, 44.11%, 48.94%, and 74.08%, respectively (Figure 3A). The CAT activity also increased by 8.98%, 12.57%, 26.94%, and 65.86%, respectively (Figure 3B). POD activity rose by 4.95%, 15.10%, 32.19%, and 57.86%, respectively (Figure 3C). SOD activity exhibited increases of 13.17%, 15.45%, 22.81%, and 35.65%, respectively (Figure 3D). The content of soluble proteins increased by 13.02%, 19.01%, 33.85%, and 50%, respectively (Figure 3E). Malondialdehyde (MDA) content increased significantly by 83.03%, 128.31%, 235.98%, and 113.56%, respectively (Figure 3F).

### 2.3. Influence of Al Toxicity on Accumulation of Several Elements in Shatian Pomelo Root

**Figure 4:**
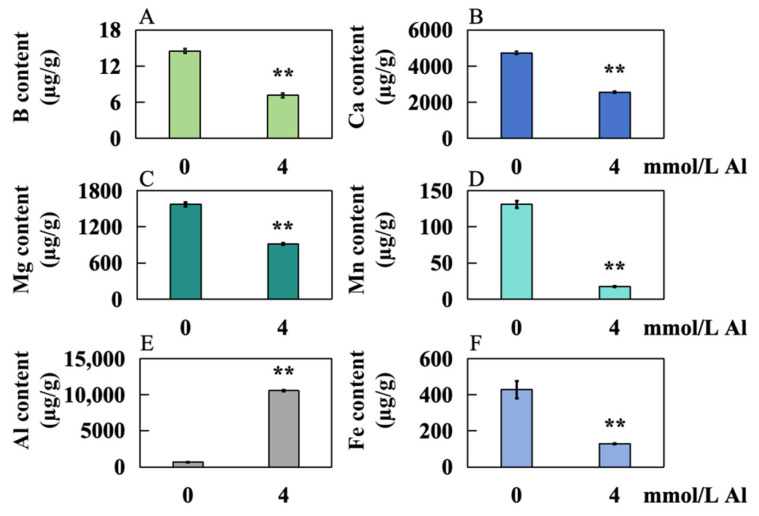
Accumulation of 6 elements in the roots of Shatian pomelo after Al toxicity. The samples were treated with 4.0 mmol/L Al for 20 days, and the control group was treated with 0 mmol/L Al. The concentrations of 6 elements were determined: (**A**) B; (**B**) Ca; (**C**) Mg; (**D**) Mn; (**E**) Al; and (**F**) Fe. The results are expressed as the mean and standard deviation of three repeated biological experiments. Student’s *t*-test was used to test the significance between the control group and the Al toxicity group. An asterisk on the bar chart indicates a significant difference (** *p* < 0.01) between the data.

Compared to the control group, the contents of B, Ca, Mg, Mn, and Fe in the experimental group decreased significantly, by 50.64%, 46.02%, 42.04%, 86.75%, and 69.92%, respectively (Figure 4A–D,F), whereas the level of Al increased significantly by 1439.25% (Figure 4E).

### 2.4. Analysis and Functional Enrichment of RNA-Seq in Shatian Pomelo Roots Under Al Stress

**Figure 5:**
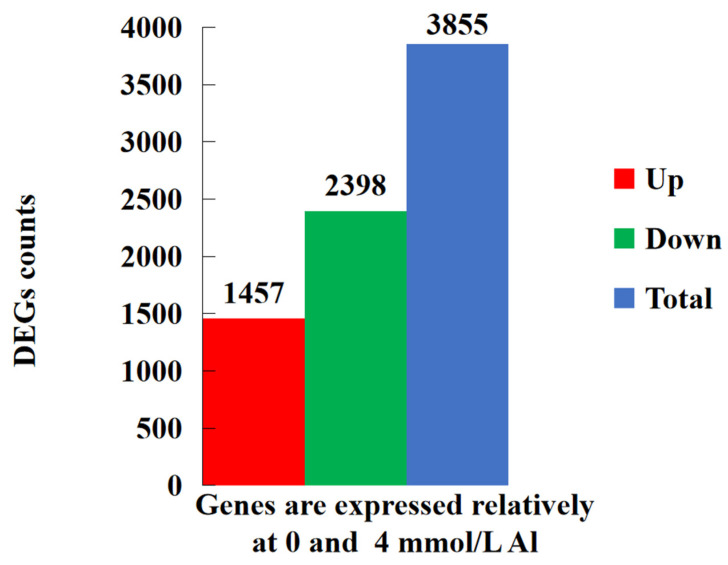
Statistical histogram of differentially expressed genes (DEGs). Blue shows the total number of DEGs (3855), green shows the number of down-regulated genes (2398), and red shows the number of up-regulated genes (1457).

Transcriptome sequencing was conducted on the roots of Shatian pomelo treated under two conditions: 0 mmol/L (control group) and 4.0 mmol/L (Al toxicity group). The sequencing produced approximately 37.5 million to 49.45 million total base sequence reads, with around 37.32 million to 49.21 million classified as clean base sequence reads. The proportion of base reads with quality scores greater than 30 (Q30) ranged from 94.14% to 94.89% (Tables S1 and S2). A total of 30,123 genes were identified in the roots of Shatian pomelo, with 3855 DEGs found in the roots treated with Al (Figure 5 and Table S3). Of these, 1457 DEGs were up-regulated, whereas 2398 DEGs were down-regulated (Figure 5 and Table S3).

**Figure 6:**
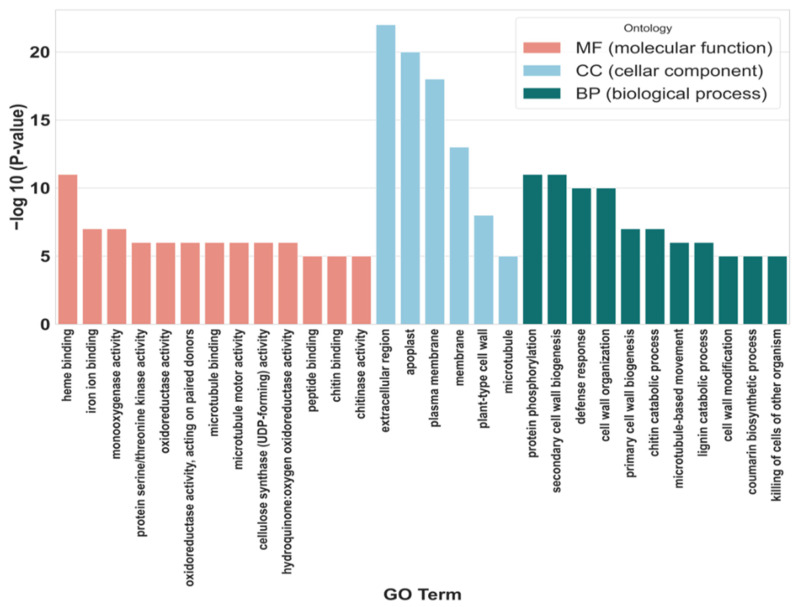
GO enrichment histogram of DEGs. Different colors in the figure indicate the categories to which different items belong: red for molecular functions (MF), blue for biological processes (BP), and green for cell components (CC). The top 30 most significantly enriched GO items in each category are displayed. The −log10 (*p* value) in the figure indicates the significance of the GO item enrichment, and the larger the value, the higher the significance of the gene enrichment.

The analysis of the DEGs in the roots of Shatian pomelo revealed significant enrichment in Gene Ontology (GO) functional categories, including biological process (BP), cellular component (CC), and molecular function (MF). The results of the enrichment analysis are presented in Figure 6 and Table S4. The DEGs were predominantly enriched in MF, followed by BP and CC. Notable concentrations of DEGs were observed in the following categories: heme binding (111 genes, 51 up-regulated and 60 down-regulated), extracellular region (192 genes, 49 up-regulated and 143 down-regulated), protein phosphorylation (163 genes, 63 up-regulated and 100 down-regulated), apoplast (117 genes, 35 up-regulated and 82 down-regulated), and plasma membrane (448 genes, 143 up-regulated and 305 down-regulated).

**Figure 7:**
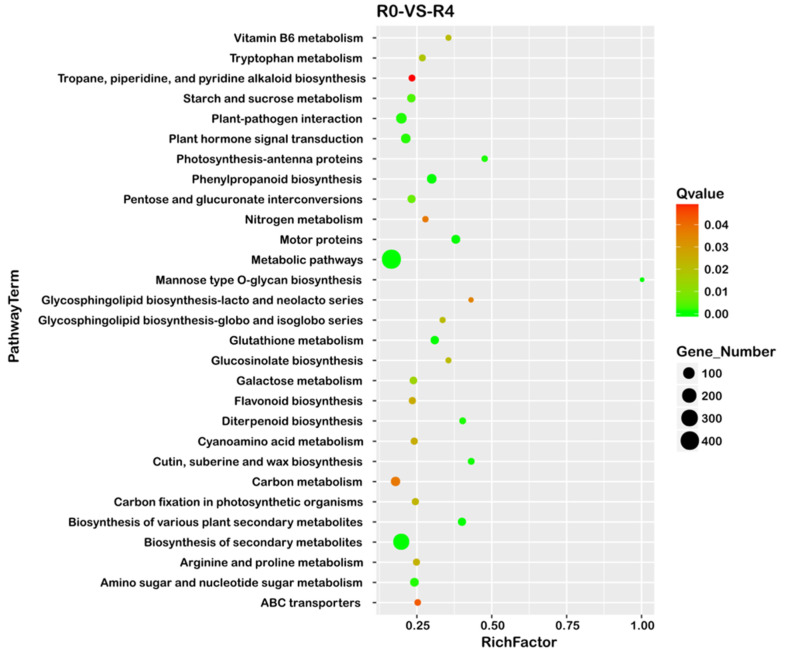
KEGG pathway enrichment map of differentially expressed genes (DEGs). PathwayTerm represents the enriched KEGG path name. RichFactor represents the ratio of the number of differential genes in a particular pathway to the total number of genes annotated in that pathway. The color of the bubbles reflects the level of enrichment significance (Q value), with green indicating higher significance (smaller Q value) and red indicating lower significance (larger Q value), and the size of the bubbles is proportional to the number of differential genes in each pathway. Only the most significant 30 pathway entries were enriched. If there were fewer than 30 pathway entries enriched, all pathway entries would be displayed.

Furthermore, KEGG pathway enrichment analysis of the DEGs in the roots of Shatian pomelo was carried out, and the results are displayed in Figure 7 and Table S5. The KEGG analysis indicated that 275 DEGs were enriched in the biosynthesis of secondary metabolites, with 112 being up-regulated and 163 down-regulated. Additionally, 38 DEGs were associated with the synthesis of motor proteins, of which 1 was up-regulated and 37 down-regulated. Two DEGs related to Mannose type O–glycan biosynthesis were both down-regulated, whereas 52 DEGs were enriched in Phenylpropanoid biosynthesis, with 22 being up-regulated and 30 down-regulated.

### 2.5. Identification of DEGs

#### 2.5.1. Identification of Hormone-Related DEGs

A total of 28 DEGs related to auxin and gibberellin synthesis were identified (Table S6). Among these, 15 DEGs were associated with auxin synthesis, with only *Cg4g008150* showing up-regulation, whereas the remaining genes were down-regulated. In terms of gibberellin (GA) synthesis, 13 DEGs were identified, with *Cg5g036390*, *Cg5g016200*, and *Cg2g044860* being the only ones up-regulated, whereas the others were down-regulated.

#### 2.5.2. Identification of Antioxidant-Related DEGs

Thirty DEGs associated with antioxidant enzymes (POD and APX) were identified (Table S7). Among these, 9 DEGs were up-regulated, whereas the remaining 21 DEGs were down-regulated.

#### 2.5.3. Identification of Ion-Transporter-Related DEGs

Sixteen DEGs associated with ion transporters were identified (Table S8). This group included four *metal tolerance protein genes*. Specifically, *Cg7g005360* and *Cg6g013150* were up-regulated, whereas *Cg9g029250* and *Cg9g029240* were down-regulated. Additionally, two *sulfate transporter genes* were identified; *Cg9g006210* was up-regulated, whereas *Cg5g035050* was down-regulated. Two *vacuolar iron transporter genes* were also observed, with *Cg2g029220* being up-regulated and *Cg3g018280* down-regulated. Furthermore, two *metal transporter genes* were noted, with *Cg3g016840* being down-regulated and *Cg1g021320* up-regulated. Among the six *calcium*-*binding protein genes*, *Cg5g040390*, *Cg2g041040*, *Cg5g010330*, and *Cg2g017370* were up-regulated, whereas Cg2g011280 and Cg2g029530 were down-regulated.

#### 2.5.4. Identification of Transcription-Factor-Related DEGs

A total of 79 DEGs associated with transcription factors were identified (Table S9). This included 25 *MYB transcription factor genes*, with 12 genes being up-regulated and 13 genes down-regulated. Furthermore, 30 *Ethylene*-*responsive transcription factor genes* were noted, of which 10 were up-regulated and the remaining 20 were down-regulated. Eight *GATA transcription factor genes* were identified, with one (*Cg1g022820*) being up-regulated and seven down-regulated. Additionally, ten *WRKY transcription factor genes* were present, with five being up-regulated and five down-regulated. Six *bHLH transcription factor genes* were found to be down-regulated.

### 2.6. Gene Expression Level Was Verified by qRT-PCR Analysis

**Figure 8:**
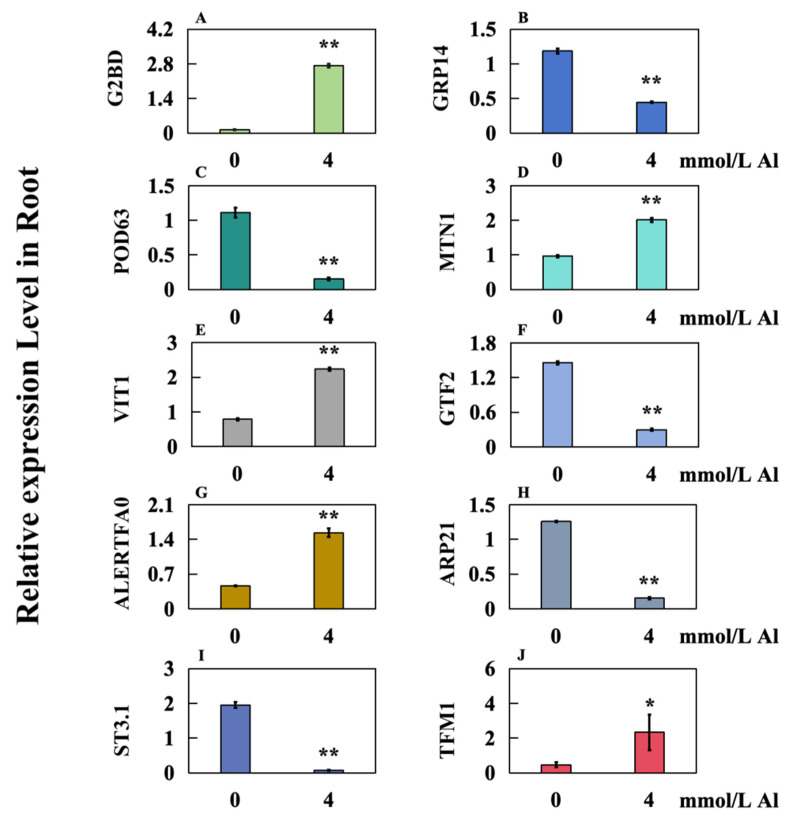
qRT-PCR detection results of 10 genes in the root system of Shatian pomelo treated with 0 mmol/L Al (control group) and 4 mmol/L Al (Al toxicity group). The results are expressed as the mean and standard deviation of three repeated biological experiments. Student’s *t*-test was used to test the significance of different concentrations between the control group and the Al toxicity group. An asterisk on the bar chart indicates a significant difference (* *p* < 0.05, ** *p* < 0.01) between the data. (**A**) *G2BD* (*Gibberellin 2-beta-dioxygenase*); (**B**) *GRP14* (*Gibberellin-regulated protein 14*); (**C**) *POD63* (*Peroxidase 63*); (**D**) *MTN1* (*Metal transporter Nramp1*); (**E**) *VIT1* (*Vacuolar iron transporter 1*); (**F**) *GTF2* (*GATA transcription factor 2*); (**G**) *ALERTFA0* (*AP2-like ethylene-responsive transcription factor At2g41710*); (**H**) *ARP21* (*Auxin-responsive protein SAUR21*); (**I**) *ST3.1* (*Sulfate transporter 3.1*); (**J**) *TFM1* (*Transcription factor MYB1*).

Three hormone-related genes were analyzed, revealing that *GRP14* and *ARP2* were extremely significantly down-regulated (Figure 8B,H), whereas *G2BD* was extremely significantly up-regulated (Figure 8A). One antioxidant oxidase-related gene, POD63, was found to be extremely significantly down-regulated (Figure 8C). Three genes related to ion transporters, including *MTN1* and *VIT1*, were extremely significantly up-regulated (Figure 8D,E), whereas *ST3.1* was extremely significantly down-regulated (Figure 8I). Regarding three transcription-factor-related genes, *GTF2* was extremely significantly down-regulated (Figure 8F), *ALERTFA0* was extremely significantly up-regulated (Figure 8G), and *TFM1* was significantly up-regulated (Figure 8J). These findings align with the results obtained from transcriptome sequencing (Table 1).

## 3. Discussion

In conditions of acidic soil, excessive Al severely inhibits plant development [24]. Al toxicity stress inhibits root elongation by limiting cell expansion, thereby inhibiting plant growth [24]. Under Al stress, root growth in plants is initially hindered [11]. Rapid inhibition of root elongation is the earliest and most significant symptom of Al toxicity, resulting in reduced and damaged roots, which limits the absorption of mineral nutrients and water, ultimately leading to reduced yields [11]. For instance, Al toxicity stress can significantly reduce the total dry biomass of *Saccharum officinarum* seedlings by 52% [25]. The root growth of Shatian pomelo was significantly inhibited under Al toxicity stress (Figure 1A–E and Figure 2A–H), and the root biomass of Shatian pomelo showed a decreasing trend with the increase in Al concentration, indicating that the root growth of Shatian pomelo was indeed severely inhibited by Al toxicity.

When plants experience severe Al toxicity stress, ROS levels rise to significantly above normal levels, leading to oxidative stress [26]. This oxidative stress triggers lipid peroxidation in the cell membrane, resulting in the production of substantial amounts of MDA [27]. In response to oxidative stress, plants activate their antioxidant defense systems, enhancing the activity of antioxidant enzymes such as SOD, POD, and APX to mitigate ROS levels and reduce oxidative damage caused by Al toxicity [28]. Consequently, the physiological condition of plants under Al toxicity stress can be effectively assessed by measuring the activities of antioxidant enzymes (SOD, POD, APX, CAT), MDA content, and soluble protein content [29]. As the concentration of the Al treatment increased, the activities of APX, CAT, POD, and SOD exhibited a progressive increase in the roots of Shatian pomelo (Figure 3A–D). This elevation in antioxidant enzyme activity indicates significant oxidative damage to the roots. Moreover, as the Al treatment concentration rose, soluble protein content also showed a trend of gradual increase, suggesting that the root cells of Shatian pomelo were responding to Al toxicity stress by accumulating more soluble proteins (Figure 3E).

The cell wall serves as a critical storage site for Al ions. The negatively charged carboxyl groups within the pectin matrix exhibit a strong binding affinity for Al^3+^, leading to substantial Al accumulation in the pectin layer of the root cell wall. This accumulation contributes to a reduction in cell wall ductility and inhibits root growth [30]. Al toxicity can severely hinder root growth in two genotypes of *Cunninghamia lanceolata*, a phenomenon also observed in *Oryza sativa*, *Arabidopsis thaliana*, and *Triticum aestivum* [31]. Under Al toxicity stress, the Al content in the roots of Shatian pomelo significantly increased, indicating that the excessive accumulation of Al may play a crucial role in inhibiting root development in this species (Figure 4E).

B plays a crucial structural support role in the cell wall of *Citrus grandis*, reducing Al binding within the cell wall and subsequently mitigating Al toxicity to cells [32]. However, in conditions of B deficiency, the structural integrity of the plant cell wall weakens, making it more susceptible to Al toxicity, which adversely affects plant growth [33]. Ca is a vital ionic component of pectin in plant cell walls; its stable presence is essential for maintaining cell wall structural stability and membrane fluidity [34]. Al interferes with the functioning of Ca channels in the plasma membrane by competing for binding sites, which inhibits Ca absorption in the roots of *Camellia sinensis* [35]. Additionally, Mg and Al also compete for similar binding sites on the cell wall and membrane in *Oryza sativa* [36]. The increase in Al content can disrupt the distribution of Mg within plant cells, further impairing Mg’s normal functions [37]. Excessive Al accumulation inhibits Mn absorption in *Triticum aestivum*, resulting in decreased Mn content [38]. In the roots of Shatian pomelo, the concentrations of B, Ca, Mg, Mn, and Fe were significantly reduced under Al toxicity stress (Figure 4A–D,F). This reduction may be closely associated with competitive absorption effects due to excessive Al accumulation in the roots and may indicate damage to the ion transport functions of the plant caused by Al toxicity. When peanut (*Arachis hypogaea*) was subjected to Al toxicity stress, it was found that there is an antagonistic relationship between Al and Mg, Ca, Mn, and other elements in the root system, possibly because Al ions occupied a large number of binding sites in the root cells of the peanut, resulting in reduced absorption of other elements [39]. In this study, the decrease in Mn content in the roots of Shatian pomelo was the most significant. This is because the massive absorption of Al in roots changes the membrane potential and changes the binding properties of cell membrane, which reduces the absorption of Mn ions by plants [40]. At the same time, Al-induced organic acid secretion may form Mn–organic acid complexes to sequester them in vacuoles, thereby inhibiting Mn absorption and transport [40]. In addition, excessive Al ions inhibited Mn ion absorption when soybean (*Glycine max*) roots responded to Mn toxicity stress [41].

Auxin is a key hormone regulating plant development and plays a critical role in various processes, including taproot growth, lateral and adventitious root formation, and root hair elongation [42]. The up-regulation of the auxin-synthesis-related gene *SAUR15* influences downstream auxin response factors *ARF6,8* and *ARF7,19*, enhancing auxin signal transmission and promoting lateral and adventitious root formation [43]. Previous studies do not indicate the expression of *SAUR21* in citrus roots under abiotic stress [43]. However, the expression of *ARP21* (*Auxin-responsive protein SAUR21*) in the roots of Shatian pomelo was found to be down-regulated under Al toxicity stress (Figure 8H). In conclusion, the down-regulated expression of *ARP21* may negatively impact auxin biosynthesis and further inhibit root growth in Shatian pomelo.

GAs are significant plant hormones that promote root elongation while inhibiting the growth of lateral and adventitious roots [44]. In response to stress, *Arabidopsis* can modulate its growth by lowering its levels of GAs and altering signaling pathways to adapt more effectively to environmental conditions [45]. This modulation primarily occurs through the down-regulation of *gibberellin regulatory protein* (*GRP*) and the up-regulation of *gibberellin-2-β dioxygenase* (*GA2ox*) [46,47]. This study observed that the expression of *gibberellin-regulated protein 14* (*GRP14*) was down-regulated (Figure 8B), whereas the expression of *gibberellin 2-beta-dioxygenase* (*G2BD*) was up-regulated in the roots of Shatian pomelo under Al toxic stress (Figure 8A). These findings suggest that the root system of Shatian pomelo may modulate the expression of gibberellin-related genes to reduce GA levels, thereby delaying root growth and enhancing resistance to Al toxicity.

Under drought conditions, tobacco (*Nicotiana tabacum*) can improve plant resistance to abiotic stress by down-regulating the expression of the *peroxidase 63* (*NtPOD63L*) gene [48]. The results showed that *POD63* was significantly down-regulated in the roots of Shatian pomelo after Al toxicity stress treatment (Figure 8C), indicating that Shatian pomelo might improve its stress resistance through this method.

Under Al stress, plants manage the chelation of Al ions in vacuoles by regulating the expression of *ion transporter* genes, which helps mitigate the harmful effects of Al [49]. For instance, the increased expression of *OsNRAMP1* in *Oryza sativa* facilitates the transport of Al into the vacuole, thereby decreasing the cytoplasmic concentration of Al and its associated toxicity [50]. Additionally, vacuolar iron transporters (VIT) have been identified as playing a crucial role in the detoxification of metal ions such as zinc, Mn, Ca, and Cu in the roots of *Arabidopsis thaliana* [51]. Following exposure to Al toxicity, the expression levels of *Metal transporter Nramp1* (*MTN1*) and *Vacuolar iron transporter 1* (*VIT1*) in the roots of Shatian pomelo were significantly up-regulated (Figure 8D,E). This indicates that Shatian pomelo may respond to Al toxicity through these two mechanisms.

Transcription factors play a significant role in the synthesis and metabolism of various substances during plant growth and can also regulate hormone signaling pathways, thereby influencing plant resilience to stress [52]. By modulating the expression of *transcription factors* such as *MYB* and *AP2/ERF*, plants can indirectly regulate the expression of downstream stress-responsive genes, enhancing their tolerance to stress [53,54]. For instance, overexpression of *RsMYB1* in *Ipomoea nil* not only significantly boosts anthocyanin synthesis in transgenic plants but also promotes the expression of genes associated with heavy metal detoxification. This enhancement allows the transgenic plants to exhibit resistance to stress from heavy metal ions such as zinc, copper, and chromium [55]. Additionally, the expressions of *TFM1* (*Transcription factor MYB1*) and *ALERTFA0* (*AP2-like ethylene-responsive transcription factor At2g41710*) were notably up-regulated in the roots of Shatian pomelo following Al toxicity stress treatment (Figure 8G,J). This indicates that the regulation by these transcription factors may influence the expression of other genes related to synthesis and metabolism, thereby improving the root tolerance of Shatian pomelo to Al stress.

**Figure 9:**
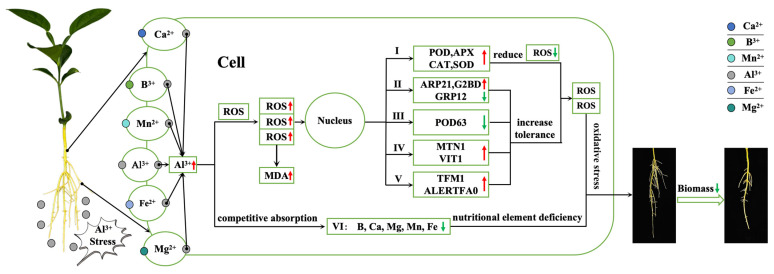
Regulatory pathway diagram of root response to Al toxicity stress in Shatian pomelo. I: antioxidant enzymes; II: genes related to hormone synthesis; III: antioxidase-related genes; IV: ion-transporter-related genes; V: transcription-factor-related genes; VI: ion. Small red arrows indicate up-regulated gene expression, increased substance content, or enzyme activity. Small green arrows indicate down-regulated gene expression or reduced substance content.

Under the stress of Al toxicity, Al will accumulate in the cell wall of the root system of Shatian pomelo, resulting in an increase in the content of intracellular reactive oxygen species (ROS). The excessive occurrence of ROS will cause oxidative stress in plants, which will increase the activity of many antioxidant enzymes (APX, CAT, POD, SOD), thereby clearing part of the ROS. Excessive reactive oxygen species also contribute to influencing gene expression in Shatian pomelo roots, such as expression of hormone, transporters, and transcription-factor-related genes (*ARP21*, *GRP14*, *G2BD*, *POD63*, *MTN1*, *VIT1*, *TFM1*, *ALERTFA0*), so as to enhance the cell tolerance of ROS; however, in the end, the active oxygen content is still higher than the normal level. Furthermore, the high concentration of Al will compete with B, Ca, Mg, Mn, and Fe for binding sites, making the content of these five ions decrease in the roots of Shatian pomelo. In the end, the root growth of Shatian pomelo will be seriously hindered and its biomass significantly decreased under the condition of ROS damage and lack of nutrition (Figure 9).

## 4. Materials and Methods

### 4.1. Plant Materials and Hydroponic Treatment

The plant material consisted of Guangxi Shatian pomelo, cultivated by Sanhao Ecological Agriculture in Public Security Town, Zhongshan County, Hezhou City, Guangxi Zhuang Autonomous Region, China. This variety was the main cultivated variety in Guangxi Province and has good genetic stability. The plants were grown in the plant culture room of the Coastal Agricultural Sciences College at Guangdong Ocean University (E: 110.30, N: 21.15). Seeds with intact seed coats and uniform sizes were selected; the outer seed coats were removed; and the soaked seeds were cultured in quartz sand for 30 days. The uniformly grown Shatian pomelo plants were then transferred to a 5 L black plastic bucket supplemented with an improved Hoagland nutrient solution for hydroponic cultivation. The hydroponic nutrient solution contained 25 µmol/L MgCl_2_, 400 µmol/L NH_4_NO_3_, 1500 µmol/L KNO_3_, 40 µmol/L Fe-EDTA (Na), 1200 µmol/L Ca(NO_3_)_2_·4H_2_O, 500 µmol/L MgSO_4_·7H_2_O, 1.5 µmol/L ZnSO_4_·7H_2_O, 0.5 µmol/L CuSO_4_·5H_2_O, 300 µmol/L (NH_4_)_2_SO_4_, 300 µmol/L K_2_SO_4_, 1.5 µmol/L MnSO_4_·H_2_O, 0.16 µmol/L (NH4)_5_MoO_24_·4H_2_O, 2.5 µmol/L NaB_4_O_7_·10H_2_O, and 500 µmol/L KH_2_PO_4_. The electrical conductivity (EC) and pH of the nutrient solution were about 1340 µs/cm and 4, respectively. All chemicals used were of analytical grade (Kermel, Tianjin, China). Al_2_(SO_4_)_3_·18H_2_O (Shanghai Reagent, Shanghai, China) served as the source of Al ions [39]. The nutrient solution was administered at concentrations of 0, 1.0, 2.0, 4.0, and 8.0 mmol/L Al for the 20-day hydroponic treatments of Shatian pomelo, with each treatment replicated three times. The Shatian pomelo plants were cultured under the following conditions: daytime temperature of 23–28 °C, night temperature of 20–22 °C, a light cycle of approximately 12 h/d, and a light intensity of 2000 lux. The nutrient solution was changed every 5 days, and, following the 20-day treatment, the roots of the Shatian pomelo were collected for data measurement.

### 4.2. Determination of Dry and Fresh Weight of Shatian Pomelo Root

The fresh root weight of each Shatian pomelo was recorded using a BS124S electronic scale (Sartorius, Göttingen, Germany). Following this, the fresh root samples were transferred to an electric blast oven (Yiheng, Shanghai, China) and dried at 75 °C for a duration of 7 days. The dry weight of each treated sample was then determined, with three biological replicates conducted [56].

### 4.3. Determination of Morphological Indexes of Shatian Pomelo Root

The roots of the Shatian pomelo were scanned using a MICROTEK MRS-9600TFU2L scanner (Microtek, Shanghai, China), capturing images of the roots at each treatment concentration. The captured images were analyzed using MICROTEK software (MICROTEK, ScanWizardEZ, version 1.0.0.1) to obtain data on various morphological parameters, including total root length, total root surface area, average root diameter, number of root tips, and total root volume. Each parameter was evaluated with three biological replicates [57].

### 4.4. Determination of Root Physiological Response Indexes of Shatian Pomelo

Following the collection of the Shatian pomelo root system, six physiological response indexes were assessed based on methods outlined by Shi et al. [39]. SOD activity was measured using the nitroblue tetrazolium method. POD activity was determined through the guaiacol method, whereas CAT activity was assessed via spectrophotometry using a Yuexi UV-5100B (Shanghai, China). Soluble protein content was quantified using the Coomassie Brilliant Blue staining technique. MDA content was measured by the thiobarbituric acid method. APX activity was analyzed following the experimental approach documented by Shi et al. [39].

### 4.5. Determination of Ion Content in Shatian Pomelo Root

Dried root samples of Shatian pomelo (0.15 g each) were collected after 20 days of treatment with 0 (control group) and 4.0 mmol/L (Al toxicity treatment group) Al. The samples were completely dissolved in 6 mL of 68% nitric acid and 0.5 mL of hydrogen peroxide (Guangzhou Reagent, Guangzhou, China). The contents of B, Mg, Al, Ca, Mn, and Fe in the Shatian pomelo roots were analyzed using an Agilent 7500cx ICP-MS (Agilent, Santa Clara, CA, USA), with three biological replicates performed [58].

### 4.6. Library Construction and Transcriptome Sequencing Analysis

Fresh root samples of Shatian pomelo seedlings were treated with 0 mmol/L (control group) and 4.0 mmol/L Al (Al toxicity treatment group) for a duration of 20 days. Twelve seedlings were treated with each concentration, and three of them were randomly selected as samples for subsequent experiments. Total RNA was extracted from these samples to construct an mRNA library, with biological replicates conducted three times for each sample. RNA extraction was performed using the Ultra-Pure RNA Kit (CWBIO, Taizhou, China). A total of 1 microgram of extracted RNA was utilized for poly(A) mRNA separation, mRNA fragmentation, cDNA synthesis, and subsequent PCR amplification and verification. The Illumina HiSeq platform of Suzhou PANOMIX Biomedical Tech Co., Ltd. (Suzhou, Jiangsu, China) was employed for mRNA library construction and sequencing. Cutadapt (V1.9.1) was used to process files in fastq format to obtain clean data, and reference genome sequences were downloaded from UCSC, NCBI, ENSEMBL, and other genomic databases. Hisat2 (v2.2.1) facilitated the alignment of clean data to the reference genome, whereas HTSeq (v0.6.1) was applied to estimate gene expression levels [41]. The DESeq2 package was utilized to identify DEGs [59]. GO terms for significantly enriched genes were determined using GOSeq (v1.34.1) [60], and the KEGG enrichment pathway analysis for DEGs was conducted using internal scripts [61]. Additional data were obtained from the integrated gene expression database, entry number PRJNA1182285.

### 4.7. qRT-PCR Detection

As per previously reported methods, ribonucleic acid (RNA) was extracted from Shatian pomelo roots utilizing a hyperpure RNA extraction kit (CWBIO, Shanghai, China). Following the removal of genomic DNA, complementary DNA (cDNA) was synthesized using a reverse transcription kit (Takara, Maebashi, Japan). Real-time fluorescence quantitative PCR (Bio-Rad Company, Hercules, CA, USA) was employed for qRT-PCR analysis [62]. The gene *Cg2g039090* (*β-tubulin*) served as a reference gene, and relative transcription levels of the target gene were calculated against this internal reference gene [63]. Primers used for qRT-PCR are shown in Table S10.

### 4.8. Data Analysis

Statistical analysis was conducted using Microsoft Excel 16.65 (Microsoft, Redmond, WA, USA) and SPSS 29.0.1.0 (IBM Corporation, New York, NY, USA). Student’s *t*-test was employed for statistical comparison and significance analysis between the two data groups, whereas the Waller–Duncan test was utilized to assess significant differences among multiple groups of data [64].

## 5. Conclusions

The effects of Al toxicity on the root system of Shatian pomelo were investigated at both physiological and molecular levels. Al toxicity disrupted the osmotic balance within and around the roots of Shatian pomelo, leading to a significant accumulation of Al in the root system. This excessive accumulation of Al heightened the activity of antioxidant enzymes, impaired the absorption and transport of other ions, disrupted normal physiological metabolism, and inhibited root development in Shatian pomelo. Furthermore, transcriptome sequencing analysis revealed 3855 DEGs in the roots of Shatian pomelo under Al stress, with 1457 genes being up-regulated and 2398 genes down-regulated. The expression of 10 DEGs—including genes associated with hormones, antioxidant enzymes, ion transport proteins, and transcription factors—was verified using quantitative real-time PCR (qRT-PCR). The verification results corroborated the findings of the transcriptome sequencing analysis. These findings enhance our understanding of the root response of Shatian pomelo to Al toxicity and provide a foundation for further exploration of the specific molecular regulatory mechanisms involved. Furthermore, the identification of Al-resistant genes can be used to carry out molecular-marker-assisted breeding or transgenic breeding, so as to accelerate the breeding process of Al-resistant Shatian pomelo varieties.

## Figures and Tables

**Table 1:** Comparison table of RNA sequencing data and qPCR results.

Gene ID	Gene Name	log2 Fold Change	qPCR Results
*Cg2g044860*	*G2BD*	3.426134	Up
*Cg6g023440*	*GRP14*	−2.06381	Down
*Cg6g023390*	*POD63*	−1.61373	Down
*Cg1g021320*	*MTN1*	1.444333263	Up
*Cg2g029220*	*VIT1*	1.235521983	Up
*Cg1g014090*	*GTF2*	−1.92661	Down
*Cg9g020390*	*ALERTFA0*	1.191315	Up
*CgUng005220*	*ARP21*	−3.664988203	Down
*Cg5g035050*	*ST3.1*	−3.63549	Down
*Cg4g018830*	*TFM1*	1.835211	Up

## Data Availability

Data are contained within the article.

## Supplementary Materials

The following supporting information can be downloaded at https://www.mdpi.com/article/10.3390/ijms252413454/s1.
