# Supplementary material for: Mechanisms of Aluminum Toxicity Impacting Root Growth in Shatian Pomelo
Source: Int J Mol Sci. 2024 Dec 15;25(24):13454. doi: 10.3390/ijms252413454 (PMC11676325; doi:10.3390/ijms252413454)
Supplement: Supplementary file 1 [file ijms-25-13454-s001.zip › TableS2-S10-12.10.pdf]

Table S2. Shatian pomelo root sample sequencing clean data quality statistics

Sample	length	Reads	Bases	Q20(%)	Q30(%)	GC(%)	N(ppm)
R0-1	146.28	38556604	5640021386	98.01	94.19	44.05	1.47
R0-2	146.19	37327090	5456976526	98.25	94.83	43.85	4.75
R0-3	146.51	44655230	6542236304	98.22	94.68	43.89	4.91
R4-1	145.98	48466406	7075296148	98.31	94.89	43.76	5.07
R4-2	146.02	48843536	7132045941	98.13	94.44	43.87	5.24
R4-3	146.2	49217248	7195357471	98.02	94.14	43.92	3.28

Table S3. Statistics of expressed genes identified in the roots of Shatian pomelo

Control-VS-Treatment	All GeneExpression	Total DEGs	Up DEGs	Down DEGs
R4-VS-R0	30123	3855	1457	2398

Table S4. GO enrichment analysis of Shatian pomelo roots

category	GO:ID	term	Up	Down	DEG	Total	pvalue	FDR
CC	GO:0005576	extracellular region	49	143	192	690	7.49E-23	4.91E-19
CC	GO:0048046	apoplast	35	82	117	360	1.64E-21	5.36E-18
CC	GO:0005886	plasma membrane	143	305	448	2091	2.59E-19	5.66E-16
CC	GO:0016020	membrane	113	190	303	1380	2.18E-14	3.57E-11
BP	GO:0009834	secondary cell wall biogenesis	0	30	30	53	2.17E-12	2.84E-09
MF	GO:0020037	heme binding	51	60	111	416	3.12E-12	3.41E-09
BP	GO:0006468	protein phosphorylation	63	100	163	655	6.36E-12	5.96E-09
BP	GO:0006952	defense response	52	64	116	448	1.3E-11	1.07E-08
BP	GO:0071555	cell wall organization	7	65	72	228	7.89E-11	5.75E-08
CC	GO:0009505	plant-type cell wall	20	54	74	263	2.88E-09	1.89E-06
MF	GO:0005506	iron ion binding	44	43	87	339	1.02E-08	6.09E-06
BP	GO:0009833	primary cell wall biogenesis	1	16	17	27	3.57E-08	1.93E-05
MF	GO:0004497	monooxygenase activity	26	26	52	170	4.16E-08	1.95E-05
BP	GO:0006032	chitin catabolic process	9	6	15	24	5.02E-08	2.2E-05
MF	GO:0016760	cellulose synthase (UDP-forming) activity	1	15	16	26	1.43E-07	5.87E-05
MF	GO:0016491	oxidoreductase activity	21	51	72	278	2.14E-07	8.24E-05
BP	GO:0007018	microtubule-based movement	0	24	24	55	2.41E-07	8.79E-05
MF	GO:0003777	microtubule motor activity	0	23	23	52	2.94E-07	0.000101
MF	GO:0004674	protein serine/threonine kinase activity	64	84	148	678	3.86E-07	0.000127
BP	GO:0046274	lignin catabolic process	0	14	14	23	4.39E-07	0.000131
MF	GO:0052716	hydroquinone:oxygen oxidoreductase activity	0	14	14	23	4.39E-07	0.000131
MF	GO:0008017	microtubule binding	1	36	37	108	5.19E-07	0.000148
MF	GO:0016705	oxidoreductase activity, acting on paired donors	20	22	42	137	6.39E-07	0.000175
MF	GO:0004568	chitinase activity	7	5	12	20	1.69E-06	0.000427
BP	GO:0009805	coumarin biosynthetic process	6	7	13	23	1.94E-06	0.000458
MF	GO:0008061	chitin binding	10	4	14	26	1.96E-06	0.000458
MF	GO:0042277	peptide binding	4	11	15	29	2.73E-06	0.000617
CC	GO:0005874	microtubule	1	39	40	131	3.27E-06	0.000715
BP	GO:0031640	killing of cells of other organism	6	2	8	12	3.95E-06	0.00081
BP	GO:0042545	cell wall modification	1	20	21	53	5.46E-06	0.001085

Table S5. KEGG analysis of DEGs in Shatian pomelo roots

Pathway ID	Pathway	Up	Down	DEGs	Total	Pvalue	Qvalue
ko04626	Plant-pathogen interaction	20	54	74	382	3.88E-05	0.000469
ko01110	Biosynthesis of secondary metabolites	112	163	275	1442	1.38E-16	9.20E-15
ko00999	Biosynthesis of various plant secondary metabolites	1	26	27	68	2.91E-09	6.44E-08
ko00750	Vitamin B6 metabolism	4	2	6	17	0.003077	0.020462
ko00515	Mannose type O-glycan biosynthesis	0	2	2	2	0	0
ko00052	Galactose metabolism	9	11	20	85	0.001604	0.013333
ko00460	Cyanoamino acid metabolism	5	10	15	63	0.004156	0.024035
ko01100	Metabolic pathways	153	282	435	2779	1.88E-11	7.69E-10
ko00330	Arginine and proline metabolism	3	11	14	57	0.003786	0.022889
ko04814	Motor proteins	1	37	38	101	2.31E-11	7.69E-10
ko02010	ABC transporters	3	7	10	40	0.008773	0.041671
ko00040	Pentose and glucuronate interconversions	3	24	27	118	0.000583	0.005173
ko00941	Flavonoid biosynthesis	10	6	16	69	0.00446	0.024717
ko01200	Carbon metabolism	21	26	47	269	0.007266	0.036659
ko04075	Plant hormone signal transduction	14	38	52	249	0.0000638	0.000652
ko00603	Glycosphingolipid biosynthesis - globo and isoglobo series	2	5	7	21	0.002793	0.020462
ko00380	Tryptophan metabolism	8	5	13	49	0.002224	0.017402
ko00500	Starch and sucrose metabolism	8	23	31	136	0.000286	0.002714
ko00480	Glutathione metabolism	20	10	30	98	5.62E-07	0.0000107
ko00966	Glucosinolate biosynthesis	6	0	6	17	0.003077	0.020462
ko00710	Carbon fixation in photosynthetic organisms	2	13	15	62	0.0035	0.022166
ko00960	Tropane, piperidine and pyridine alkaloid biosynthesis	8	4	12	52	0.01072	0.049166
ko00520	Amino sugar and nucleotide sugar metabolism	17	18	35	147	0.0000498	0.000552
ko00940	Phenylpropanoid biosynthesis	22	30	52	176	3.88E-10	1.03E-08
ko00910	Nitrogen metabolism	2	6	8	29	0.007442	0.036659
ko00073	Cutin, suberine and wax biosynthesis	4	8	12	28	0.0000114	0.000189
ko00601	Glycosphingolipid biosynthesis - lacto and neolacto series	0	3	3	7	0.006441	0.034269
ko00196	Photosynthesis - antenna proteins	0	9	9	19	0.0000307	0.000408
ko00904	Diterpenoid biosynthesis	4	8	12	30	0.0000285	0.000408

Table 6. DEGs of phytohormone signal transduction pathways in Shatian pomelo roots under Al stress

GeneID	log2FoldChange	Description
Cg4g018030	-1.62641	Auxin-responsive protein SAUR76
CgUng005220	-3.66499	Auxin-responsive protein SAUR21
Cg9g006770	-1.26442	Auxin-responsive protein IAA14
Cg4g008150	2.59936	Auxin-responsive protein IAA1
Cg5g034600	-3.33676	Auxin-induced protein AUX28
CgUng005210	-2.99963	Auxin-induced protein 6B
Cg5g034610	-2.51601	Auxin-induced protein 22D
Cg3g023800	-1.57232	Auxin-induced protein 15A
Cg8g001890	-1.90732	Auxin transporter-like protein 5
Cg3g016080	-2.00636	Auxin transporter-like protein 3
Cg6g006550	-1.36538	Auxin transporter-like protein 2
Cg6g023900	-1.64229	Auxin efflux carrier component 8
Cg3g017880	-1.42049	Auxin efflux carrier component 6
Cg2g033780	-1.01611	Auxin efflux carrier component 3
Cg4g020810	-7.1358	Auxin efflux carrier component 2
Cg3g020210	-1.13762	Gibberellin-regulated protein 9
Cg6g018040	-1.1673	Gibberellin-regulated protein 5
Cg6g024880	-1.84244	Gibberellin-regulated protein 4
Cg6g023440	-2.06381	Gibberellin-regulated protein 14
Cg5g036390	1.133611	Gibberellin receptor GID1B
Cg1g016030	-2.29925	Gibberellin 3-beta-dioxygenase 1
Cg2g040470	-1.92798	Gibberellin 2-beta-dioxygenase 8
Cg5g038810	-2.39878	Gibberellin 2-beta-dioxygenase 2
Cg5g016200	3.48885	Gibberellin 2-beta-dioxygenase 1
Cg2g044860	3.426134	Gibberellin 2-beta-dioxygenase
Cg9g023410	-6.41779	Gibberellin 20-oxidase-like protein
Cg5g042980	-2.34422	Gibberellin 20 oxidase 2
Cg9g026010	-1.875	Gibberellin 20 oxidase 1

Table S7. Identification of DEGs associated with antioxidant enzymes in roots of Shatian pomelo under Al stress

Gene ID	log2FoldChange	Description
Cg5g023710	-2.97222	Probable peroxidase 61
Cg7g009540	-1.16837	Peroxidase N1
Cg2g001490	-2.30694	Peroxidase N
Cg2g001460	1.287111	Peroxidase C3
Cg2g001430	1.085203	Peroxidase C3
Cg3g023260	-1.80925	Peroxidase 9
Cg7g014170	-1.15621	Peroxidase 65
Cg1g001240	-1.33095	Peroxidase 64
Cg3g024510	-3.07489	Peroxidase 64
Cg6g023390	-1.61373	Peroxidase 63
Cg5g031940	-3.11891	Peroxidase 57
Cg2g008860	-1.33586	Peroxidase 52
Cg9g024340	-6.3249	Peroxidase 52
Cg1g022560	-9.96207	Peroxidase 52
Cg1g006370	1.087595	Peroxidase 51
Cg5g039010	-4.36331	Peroxidase 5
Cg5g039000	-4.56015	Peroxidase 5
Cg9g001000	2.289721	Peroxidase 40
Cg9g003710	-3.73152	Peroxidase 3
Cg2g018030	-5.72879	Peroxidase 27
Cg1g009400	1.344082	Peroxidase 21
Cg2g001440	1.121688	Peroxidase 15
Cg2g001380	-1.31386	Peroxidase 15
Cg7g015380	-1.04955	Peroxidase 11
Cg8g005280	1.274362	Peroxidase 10
Cg1g011710	-4.90056	Lignin-forming anionic peroxidase
Cg1g011700	-7.44764	Lignin-forming anionic peroxidase
Cg3g016700	1.197089	L-ascorbate peroxidase T, chloroplastic
Cg1g022550	8.273938	Cationic peroxidase 1
Cg6g010260	-9.12255	Cationic peroxidase 1

Table S8. Identification of DEGs related to ion transport in roots of Shatian pomelo under Al stress

geneID	log2FoldChange	Description
Cg7g005360	1.841515	Metal tolerance protein 11
Cg6g013150	1.632734	Metal tolerance protein 4
Cg9g029250	-4.42041	Metal tolerance protein 10
Cg9g029240	-1.96516	Metal tolerance protein 10
Cg5g035050	-3.63549	Sulfate transporter 3.1
Cg9g006210	4.911366	Sulfate transporter 3.1
Cg3g018280	-1.49539	Vacuolar iron transporter homolog 3
Cg2g029220	1.235522	Vacuolar iron transporter 1
Cg3g016840	-2.76818	Metal transporter Nramp5
Cg1g021320	1.444333	Metal transporter Nramp1
Cg5g040390	1.285098	Calcium-binding protein KIC
Cg2g041040	1.500081	Calcium-binding protein KRP1
Cg5g010330	1.887995	Probable calcium-binding protein CML46
Cg2g011280	-1.46727	Putative calcium-binding protein CML19
Cg2g029530	-2.10942	Probable calcium-binding protein CML25
Cg2g017370	1.526463	Probable calcium-binding protein CML47

Table S9. Identification of DEGs related to transcription factor in roots of Shatian pomelo under Aluminum stress

geneID	log2FoldChange	Description
Cg9g022730	-4.40413	Transcription factor MYBS2
CgUng002190	-1.05533	Transcription factor MYBS1
Cg3g014950	-2.84676	Transcription factor MYB93
Cg5g029630	-1.36104	Transcription factor MYB93
Cg5g013190	-1.49689	Transcription factor MYB86
Cg5g005500	-1.19182	Transcription factor MYB86
Cg7g023280	-1.23002	Transcription factor MYB83
Cg8g003880	-1.35537	Transcription factor MYB73
Cg3g021810	-1.40141	Transcription factor MYB73
Cg5g022560	1.653431	Transcription factor MYB62
Cg5g039420	-1.56485	Transcription factor MYB61
Cg2g037000	-1.60514	Transcription factor MYB61
Cg8g021710	-1.23579	Transcription factor MYB59
Cg9g008130	1.901769	Transcription factor MYB4
Cg8g010070	1.017877	Transcription factor MYB4
Cg5g017180	1.654944	Transcription factor MYB36
Cg5g040470	1.016164	Transcription factor MYB3
Cg2g012470	1.056788	Transcription factor MYB27
Cg2g036140	3.643	Transcription factor MYB2
Cg5g038840	1.052437	Transcription factor MYB2
Cg4g014320	1.788013	Transcription factor MYB1R1
Cg2g041090	1.032861	Transcription factor MYB14
Cg9g000740	1.915272	Transcription factor MYB108
Cg5g010420	-2.28467	Transcription factor MYB106
Cg4g018830	1.835211	Transcription factor MYB1
Cg2g009690	1.939246	Ethylene-responsive transcription factor RAP2-6
Cg9g012290	-1.83125	Ethylene-responsive transcription factor RAP2-4
Cg5g001420	-1.50015	Ethylene-responsive transcription factor RAP2-4
Cg2g000460	-4.4143	Ethylene-responsive transcription factor RAP2-11
Cg6g025280	-1.66543	Ethylene-responsive transcription factor RAP2-11
Cg5g000120	1.126089	Ethylene-responsive transcription factor ERN2
Cg8g004600	-4.91478	Ethylene-responsive transcription factor ERF115
Cg1g026460	3.993738	Ethylene-responsive transcription factor ERF110
Cg7g011250	1.453024	Ethylene-responsive transcription factor ERF110
Cg2g014260	-1.50068	Ethylene-responsive transcription factor ERF053
Cg3g016320	-2.26966	Ethylene-responsive transcription factor ERF036
Cg5g044100	-2.44486	Ethylene-responsive transcription factor ERF034
Cg4g015400	-1.25294	Ethylene-responsive transcription factor ERF023
Cg5g040600	-1.70604	Ethylene-responsive transcription factor ERF023
Cg5g021590	-1.92555	Ethylene-responsive transcription factor ERF022

Cg4g017840	1.626659	Ethylene-responsive transcription factor ERF016
Cg4g001690	-1.56662	Ethylene-responsive transcription factor ERF003
Cg1g026480	2.34784	Ethylene-responsive transcription factor ABR1
Cg9g007920	1.360357	Ethylene-responsive transcription factor 6
Cg9g022820	-1.22349	Ethylene-responsive transcription factor 5
Cg2g042530	-1.32109	Ethylene-responsive transcription factor 4
Cg9g022790	2.499133	Ethylene-responsive transcription factor 2
Cg9g007940	4.103726	Ethylene-responsive transcription factor 1B
Cg5g044300	-3.80958	Ethylene-responsive transcription factor 13
Cg5g024000	-5.83883	Ethylene-responsive transcription factor 13
Cg2g013290	-1.004	Ethylene-responsive transcription factor 12
Cg5g044360	-2.04676	Ethylene-responsive transcription factor 1
Cg2g003030	-2.18559	AP2-like ethylene-responsive transcription factor PLT2
Cg6g000260	-1.1566	AP2-like ethylene-responsive transcription factor BBM1
Cg9g020390	1.191315	AP2-like ethylene-responsive transcription factor At2g41710
Cg7g006680	-1.34248	GATA transcription factor 11
Cg5g031000	-1.10147	GATA transcription factor 1
Cg1g014090	-1.92661	GATA transcription factor 2
Cg5g043880	-2.51398	GATA transcription factor 2
Cg1g022820	3.926498	GATA transcription factor 5
Cg4g006690	-2.88903	GATA transcription factor 21
Cg1g004300	-1.37536	GATA transcription factor 18
Cg4g004250	-2.78649	GATA transcription factor 12
Cg6g008950	-1.45325	Transcription factor bHLH76
Cg1g008260	-2.41504	Transcription factor bHLH94
Cg3g013130	-1.14893	Transcription factor bHLH77
Cg6g018550	-6.24028	Transcription factor bHLH84
Cg5g039930	-1.80248	Transcription factor bHLH57
Cg5g035630	-1.26948	Transcription factor BHLH42
Cg6g024200	-2.81943	WRKY transcription factor 72A
Cg5g003350	-2.31918	WRKY transcription factor 72A
Cg5g041490	-1.5543	WRKY transcription factor 22
Cg7g016740	2.453615	Probable WRKY transcription factor 75
Cg2g010670	2.907952	Probable WRKY transcription factor 75
Cg7g010390	1.508024	Probable WRKY transcription factor 75
Cg5g041440	-1.92154	Probable WRKY transcription factor 75
Cg4g019190	2.030446	Probable WRKY transcription factor 50
Cg1g021340	1.730282	Probable WRKY transcription factor 40
Cg9g027680	-1.61434	Probable WRKY transcription factor 27

Table S10. Primers used for qPCR in the study

Gene ID	Gene name	Forward Primer (5' - 3')	Reverse Primer (5' - 3')
Cg6g023440	Gibberellin-regulated protein 14 (GRP14)	GGTCAAACAAAGATTGCATCC	CTCTGTTGCCATAAGTTCCTG
CgUng005220	Auxin-responsive protein SAUR21 (ARP21)	ACACTTAGCTGTCTATGTCGG	AACTCCTCTTCTGCCTGACTC
Cg2g044860	Gibberellin 2-beta-dioxygenase (G2BD)	CAAGAACGCTCACAGAAATTCC	CTGCCATCAATTCCATTACCTG
Cg6g023390	Peroxidase 63(POD63)	CAATCACCAACAAGCAAATCAC	GAGGTTATGAGGATGGAGGAG
Cg5g035050	Sulfate transporter 3.1 (ST3.1)	GTCGCCTCACTTCTCATAGC	GATAAACCCTAGCCTTAGCAGTC
Cg2g029220	Vacuolar iron transporter 1(VIT1)	TCCTCCATCATTCTCATCGCT	TCTTCTTGTTCTCGCTTTAGTTCC
Cg1g021320	Metal transporter Nramp1 (MTN1)	GTCTCTAGCAGCCAATCTCG	AGAAGAACACCAATCCACACTG
Cg1g014090	GATA transcription factor 2 (GTF2)	CTAACTCCACTGATTCTTCCAC	AAAGCCATTCTAACTCTGCC
Cg9g020390	AP2-like ethylene-responsive transcription factor At2g41710 (ALERTFA0)	CCACTTCTGACCAACTATAACAC	TGCTCTCAGTCTTCGTAACC
Cg4g018830	Transcription factor MYB1 (TFM1)	CTTGGAAAGCCGTTGAAGAC	TCCCGCATCTCTTAAGACCT
Cg2g039090	β-tubulin	TGGATGTTGTTAGAAAGGAAGCTG	GATACCTTAGGAGACGGGAAGAC
